# Learning hierarchically organized science categories: simultaneous instruction at the high and subtype levels

**DOI:** 10.1186/s41235-019-0200-5

**Published:** 2019-12-19

**Authors:** Robert M. Nosofsky, Colin Slaughter, Mark A. McDaniel

**Affiliations:** 1 Psychological and Brain Sciences, Indiana University, 1101 E. Tenth Street, Bloomington, IN 47405 USA; 2 Washington University in St. Louis, St. Louis, MO USA

**Keywords:** Category learning, Education, Instruction, Memory

## Abstract

**Background:**

Most science categories are hierarchically organized, with various high-level divisions comprising numerous subtypes. If we suppose that one’s goal is to teach students to classify at the high level, past research has provided mixed evidence about whether an effective strategy is to require simultaneous classification learning of the subtypes. This past research was limited, however, either because authentic science categories were not tested, or because the procedures did not allow participants to form strong associations between subtype-level and high-level category names. Here we investigate a two-stage response-training procedure in which participants provide both a high-level and subtype-level response on most trials, with feedback provided at both levels. The procedure is tested in experiments in which participants learn to classify large sets of rocks that are representative of those taught in geoscience classes.

**Results:**

The two-stage procedure yielded high-level classification performance that was as good as the performance of comparison groups who were trained solely at the high level. In addition, the two-stage group achieved far greater knowledge of the hierarchical structure of the categories than did the comparison controls.

**Conclusion:**

In settings in which students are tasked with learning high-level names for rock types that are commonly taught in geoscience classes, it is best for students to learn simultaneously at the high and subtype levels (using training techniques similar to the presently investigated one). Beyond providing insights into the nature of category learning and representation, these findings have practical significance for improving science education.

## Significance

A fundamental part of science education involves teaching the categories of the target domain. Furthermore, in numerous cases, the categories are hierarchically organized, with high-level divisions broken down into fundamental subtypes. This research addresses the question whether requiring students to learn the subtypes may sometimes lead to more effective teaching of the high-level divisions themselves. The question is pursued here in basic-research laboratory experiments that investigate performance in a real-world science domain; namely, rock classification in the geologic sciences. In particular, the participants in our studies learn to classify sets of images of rocks into categories that are commonly taught in college-level introductory geoscience courses. The results from the work provide firm suggestions for methods that are likely to be effective for teaching the hierarchical structure of categories in the science classroom.

## Introduction

An integral part of science education is learning the categories of the domain of interest. For instance, botany focuses on classifying and learning plants, entomology on classifying and learning insects, and geology on classifying and learning rocks. As argued below, learning these categories is fundamental to scientific reasoning and inference and forms a significant component of college-level science curricula.

In the present research, our example target domain is rock classification in the geologic sciences. As is true of numerous natural science categories, rock types have a graded structure, with clear prototypical instances at their centers, but also with many less typical instances (Rosch, 1973; Smith & Medin, 1981). Thus, individual samples of the same type of rock can often display remarkable within-category variability. In addition, as is also true of most natural categories, the boundary lines dividing different rock types are often fuzzy, and the distributions of members from contrasting categories may sometimes even overlap. Moreover, rock categories have a hierarchical structure in which broader level categories (igneous, metamorphic, sedimentary) subsume lower level subtypes organized within each broad-level category (as displayed in Table 1). In the senses described above, rock classification appears to be both a challenging and representative example of natural science category learning.

**Table 1:** A breakdown of the rock high levels and subtype levels used in the study

Igneous	Metamorphic	Sedimentary
Andesite	Amphibolite	Bituminous coal
Basalt	Anthracite	Breccia
Diorite	Gneiss	Chert
Gabbro	Hornfels	Conglomerate
Granite	Marble	Dolomite
Obsidian	Migmatite	Micrite
Pegmatite	Phyllite	Rock Gypsum
Peridotite	Quartzite	Rock Salt
Pumice	Schist	Sandstone
Rhyolite	Slate	Shale

Teaching rock classifications is one of the early goals in geoscience education. Introductory college-level geology textbooks devote multiple chapters to the classification of rocks (e.g., Marshak, 2015; Tarbuck & Lutgens, 2017), as does the National Association of Geoscience Teachers/American Geological Institute *Laboratory manual in physical geology* (Cronin, 2018). The textbook and laboratory manual chapters provide detailed descriptions of the major categories of rocks, and they attempt to characterize the key features and dimensions that organize and compose the rock categories. Further, laboratory sessions and field work associated with college-level introductory geoscience courses often devote significant amounts of time to the training of rock classifications.

Teaching fundamental categories, such as rock types in geology, is a core component of science curricula for a good reason. Categories are the building blocks of our basic thought processes and they provide an efficient means to allow us to reason about the nature of the world and draw inferences. Examples of the important role of rock classification in reasoning and inference abound in geology. For example, as conceptualized in the geologic sciences, one of the broad high-level divisions of rocks is the class of igneous rocks; this high-level division is composed of rocks formed from the solidification of magma. A major distinction between categories of igneous rocks is that of intrusive versus extrusive rocks. Intrusive igneous rocks, such as granite, are formed when magma solidifies at depth. In this case, the magma cools slowly, allowing large crystalline mineral structures to develop resulting in a coarse grain. By contrast, extrusive igneous rocks, such as rhyolite, are formed when magma solidifies in a surface environment. In this case, the magma cools quickly, resulting in a fine-grained crystalline structure. A geologist examining a terrain might therefore obtain clues about its history by determining whether the rocks that compose the terrain are intrusive or extrusive igneous rocks as evidenced by the grain size of the rocks.

As alluded to above, in numerous scientific domains the categories are hierarchically organized. For example, geologic scientists divide rocks into three, broad, high-level categories: igneous, metamorphic and sedimentary (Marshak, 2015; Tarbuck & Lutgens, 2017). These broad categories are defined by how the rocks are formed. In brief, whereas igneous rocks are formed from the solidification of magma, metamorphic rocks are formed when other rocks are exposed to extreme heat and pressure, causing them to undergo changes in their physical or chemical structure. Finally, sedimentary rocks are formed when mineral and organic particles are deposited on the floor of bodies of water and are eventually cemented together. However, each of these broad, high-level divisions is broken down into fundamental subtypes. For example, common subtypes of igneous rocks are granite, obsidian and pumice; common subtypes of metamorphic rocks are gneiss, marble and quartzite; and common subtypes of sedimentary rocks are sandstone, shale and limestone.

If we suppose that the goal of the instructor is to teach students to classify into the high-level divisions of igneous, metamorphic and sedimentary, then a reasonable hypothesis is that, to achieve that goal, it might be best to focus training on that high level, without also requiring that students learn to discriminate among all the subtypes. Consistent with the principle of transfer-appropriate processing (e.g., Blaxton, 1989; Thomas & McDaniel, 2007), such training would focus on the outcome that is the instructor’s primary goal. Recent work reported by Noh, Yan, Vendetti, Castel, and Bjork (2014) is consistent with this hypothesis. In one of their conditions, these researchers had participants learn to classify pictures of snakes into the high-level categories of venomous versus nonvenomous. The participants’ high-level classification performance was better if they focused their learning solely at that high level, rather than also being required to simultaneously learn to discriminate among different subtypes of the venomous and nonvenomous snakes. Presumably, by focusing on the high level, participants learned more effectively to attend to features that are highly diagnostic of membership in the contrasting high-level categories (Nosofsky, 1986; Shepard, Hovland, & Jenkins, 1961). For example, venomous snakes tend to have arrow-shaped heads whereas nonvenomous snakes tend to have spoon-shaped heads. By contrast, use of the head-shape feature does not allow one to discriminate among different subtypes of venomous snakes or different subtypes of nonvenomous ones.

However, other research has sometimes pointed in the opposite direction, with subtype-level training being shown to be beneficial. For example, using a particular artificially designed category structure, several researchers found that learners displayed more accurate classification when trained and tested at a subtype level of a hierarchy than at a higher, more general level (Lassaline, Wisniewski, & Medin, 1992; Palmeri, 1999; Verheyen, Ameel, Rogers, & Storms, 2008).

**Fig. 1:**
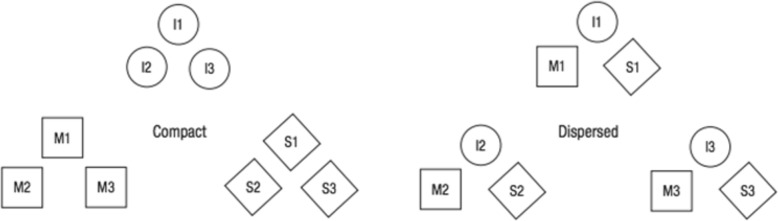
A schematic illustration of the compact and dispersed category structures tested in Nosofsky et al.’s (2017) study. Circles indicate igneous, squares indicate metamorphic, and diamonds indicate sedimentary categories

Nosofsky, Sanders, Gerdom, Douglas, and McDaniel (2017) found evidence consistent with the hypothesis that the best teaching strategy may vary depending on the structure of the categories being learned. Using real-world rock categories as their target domain, these researchers compared two different teaching strategies across two different category structures. One teaching strategy focused solely on teaching the high-level divisions whereas, in the second, participants simultaneously learned to classify at both the high and subtype levels (see below for further details). The to-be-learned category structures, illustrated schematically in Fig. 1, were either *compact* or *dispersed*. As shown in Fig. 1, in both category-structure conditions each high-level division (igneous, metamorphic and sedimentary) was composed of three subtypes of rocks. In the compact condition, the subtypes were chosen such that all three subtypes belonging to the same high-level division were highly similar to one another, while being dissimilar to the subtypes from the alternative high-level categories. Thus, each high-level category formed a relatively compact cluster in a multidimensional similarity space. By contrast, in the dispersed condition, the subtypes were chosen such that each of the three subtypes belonging to the same high-level division were *dissimilar* to one another, occupying separate clusters of the similarity space. At the same time, each subtype was similar to individual subtypes from both other high-level divisions. We acknowledge that readers may find the category structure in the dispersed condition to be contrived because three different categories with the same high-level name are clustered together within each of the similarity groups. Nevertheless, we emphasize here that the category structure was produced by sampling *real-world* subtypes from the *actual* high-level divisions of igneous, metamorphic and sedimentary rocks. In other words, in the real world, there are many cases, for example, of igneous subtypes that are highly similar to metamorphic subtypes, and of igneous subtypes that are highly dissimilar from one another. We expand on this point in greater detail below.

In brief, Nosofsky et al. (2017) found that learning the high-level names of the rocks in the compact condition was better when the training procedure focused solely on teaching the high-level names for the rocks. By contrast, learning the high-level names of the rocks in the dispersed condition was better when the training procedure required participants to simultaneously learn both the high-level and subtype-level names. The latter result is of potentially high practical significance. Nosofsky et al. (2017) and Nosofsky, Sanders, Meagher, and Douglas (2018) conducted extensive similarity-scaling studies in which participants rated the similarity among pairs of items drawn from a large battery of igneous, metamorphic and sedimentary rocks that are representative of those taught in college-level introductory geoscience classes. Multidimensional scaling analyses of the similarity-judgment data revealed that the structure of the igneous, metamorphic and sedimentary categories does in fact appear to be relatively disorganized and dispersed (although not to the extreme degree illustrated in the right panel of Fig. 1).

Although suggestive, there were at least two major limitations of Nosofsky et al.’s (2017) initial study in terms of its practical implications. First, as already noted, the researchers constructed the compact and dispersed conditions by selectively sampling rock subtypes from the three high-level categories. A natural question is how the alternative teaching strategies would fare if participants were tasked with classifying a larger set of ‘authentically’ sampled rocks that are more representative of those taught in introductory geoscience classes.

A second and more fundamental limitation concerns the detailed method that Nosofsky et al. (2017) used in the condition in which participants learned to classify at both the high and subtype levels simultaneously. In particular, Nosofsky et al. used a ‘simultaneous paired-naming’ procedure, in which the response alternatives associated with each rock consisted simultaneously of the high-level category and the subtype name. For example, one rock might be designated as ‘igneous-granite’ and another as ‘metamorphic-marble’. Both members of the paired name were always simultaneously present when participants made their responses. To measure high-level naming performance, the researchers scored a response as correct if the participant indicated the correct high-level name, regardless of the subtype-level response that was indicated. Unfortunately, however, although the procedure was well motivated from a theoretical standpoint (see formal modeling presented by Nosofsky et al., 2017), from a practical standpoint it does not allow one to determine whether participants actually learned the high-level names at all. In particular, a participant could have learned at least some rocks solely at the subtype-naming level, without ever establishing an association between the subtype name and the high-level name. For example, if a participant learned that a particular rock sample was granite, then he or she would press the ‘igneous-granite’ response key and receive credit towards a correct high-level categorization response. It is unknown, however, whether the participant could have correctly classified the sample as ‘igneous’ if the subtype name (‘granite’) was not simultaneously present.

Miyatsu, Nosofsky, and McDaniel (in press) conducted a series of experiments to begin to address both of the above-stated limitations. One change to Nosofsky et al.’s (2017) experiment was that, rather than using the selectively sampled compact and dispersed structures, Miyatsu et al. had participants learn a larger number of rock subtypes that provided a more representative sampling of the sets of igneous, metamorphic and sedimentary rocks found in the natural world. A second change involved the training and testing procedures in the conditions in which participants were trained to classify at both levels of the rock category hierarchy. Of most direct relevance to the current practical question were the procedures used by Miyatsu et al. (in press) in their experiments 1 and 2. In their experiment 1, Miyatsu et al. used an observational training procedure in which participants studied pictures of rocks with names assigned to them. One group of participants studied the pictures with just the high-level names, whereas the second group studied the pictures with both the high-level and subtype names. (During this observational training participants were provided with general instructions to learn the names associated with the rocks.) Following the observational training phase, participants were tested on their ability to classify both old and new rock pictures into their high-level categories. Participants in the high-level name-only training group performed significantly better at the time of the test (on both old and new items) than did participants in the paired-name training group. One limitation of this design, however, involves the problem that many participants in the paired-name training group may have focused their learning on the subtype names without ever forming associations between the subtype-level and high-level names. Clearly, such participants would then be severely impaired when tested on their ability to classify the items into their high-level categories.

To potentially address this limitation, in a second experiment Miyatsu et al. (in press) tested two new groups of participants. The first group was again trained using observational training of only the high-level names, and the participants knew that they would eventually be tested on their ability to produce the correct high-level classifications. A second group, however, first engaged in observational classification training at only the *subtype* level (and were instructed to learn the subtype-level name assignments). Next, the participants in this group engaged in a separate paired-associate training phase in which they were trained on the pairings between the subtype-level and high-level category names. Finally, during the subsequent test phase, participants in this group attempted to classify each rock into its high-level category. Just as in their experiment 1, Miyatsu et al. found that the group that was trained on only the high-level names performed significantly better in high-level classification at the time of the test than did the participants in the subtype-level/paired-associate training group. Unfortunately, however, this new experimental design ended up with essentially the same main limitation as the previous one: during the paired-associate training phase, participants in Miyatsu et al.’s study achieved an accuracy level of only .76 in producing the high-level category name associated with each subtype name. Thus, even if participants had learned to classify extremely accurately at the subtype level, it stands to reason that the high-level classification performance for this group (during the final test phase) would be impaired. In addition, Miyatsu et al.’s experiment 2 design also had the limitation that, for the subtype-level/paired-associate group, no form of high-level training occurred in the initial classification-learning phase (participants were trained at *only* the subtype level). Thus, there was no opportunity for participants to learn to give greater attention to features that were diagnostic at the high level of classification.^1^

The central motivation of our present research was to continue to investigate the potential utility of subtype-level training in improving high-level classification in this rock domain. Our main goal was to try to develop a training procedure that maintained the potential advantages of simultaneous high-level and subtype-level training, while directly addressing the limitation that many participants may fail to learn associations between the subtype-level and high-level names. To preview, we introduce a new condition in which classification training again takes place simultaneously at both the high and subtype levels, but which places stronger emphasis than the previous studies on the goal of learning *both* levels, and which provides continuous practice throughout training to promote the achievement of this goal. We compare the performance of this new training group to that of two comparison groups who are trained using procedures similar to those in the previous studies of Nosofsky et al. (2017) and Miyatsu et al. (in press).

## Experiment 1A

Across three different conditions, participants learned to classify images of rocks into the high-level categories of igneous, metamorphic and sedimentary. In all conditions, the instructions to the participants emphasized that their primary task was to learn these high-level category assignments. In some of the conditions, the participants also learned to classify the rocks into their subtype categories. The complete set of rocks comprised 30 subtypes, 10 subtypes from each of the three high-level categories. The subtypes are listed in Table 1. The subtypes are highly representative of those that are commonly taught in introductory college-level geoscience classes, and are among the major ones listed and described in introductory textbooks (e.g., Marshak, 2015; Tarbuck & Lutgens, 2017). Because it was unrealistic to expect a participant to learn all 30 subtypes in a single 1-h session, each individual participant was randomly assigned 15 of the 30 subtypes to learn (five from each of the three high-level categories).^2^

**Fig. 2:**
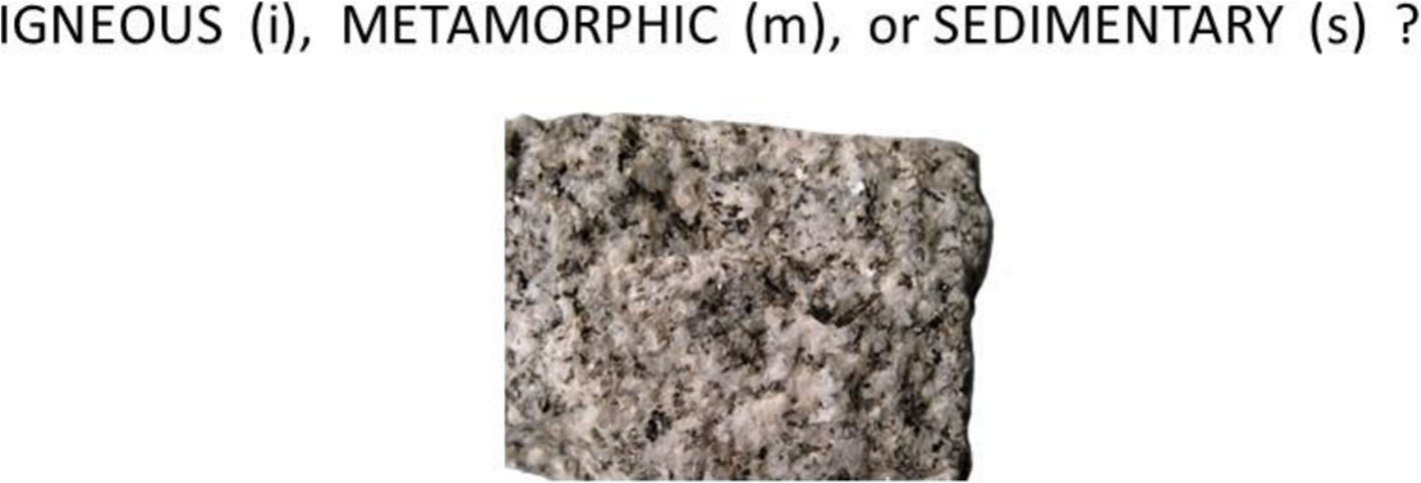
Schematic example of a trial from the training and test phases of condition 1 and the test phase of condition 3

In condition 1, participants were trained on only the high-level names of the rocks. An example screenshot of the question prompt on a typical trial is presented in Fig. 2. As illustrated, on each trial, an individual rock would be presented, and the participant would attempt to classify it into one of the three high-level categories. Feedback was provided only with respect to the high-level category to which the rock belonged. In the test phase, participants continued to classify items at only this high-level of categorization.

**Fig. 3:**
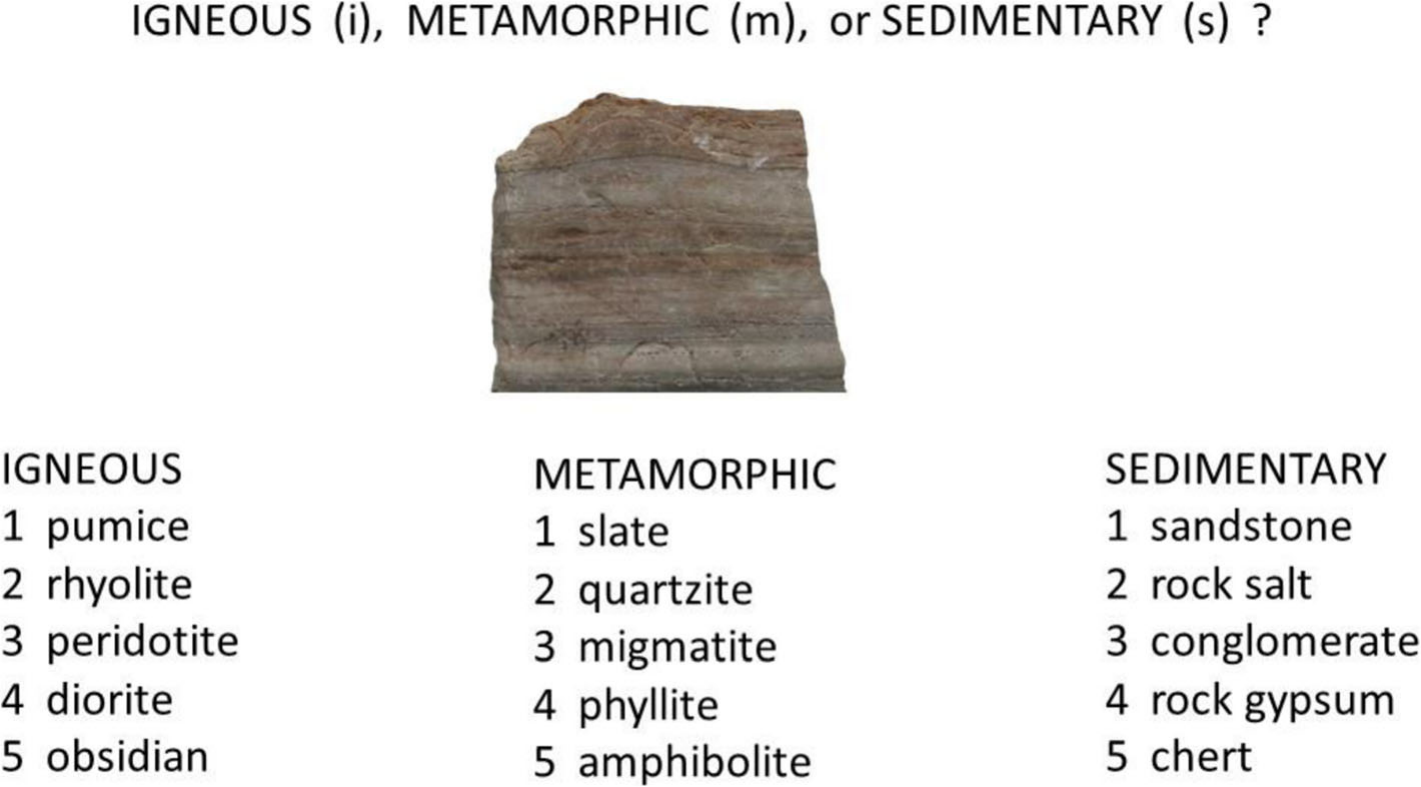
Schematic example of the first stage of a typical trial from the training and test phases of condition 2 and the training phase of condition 3

**Fig. 4:**
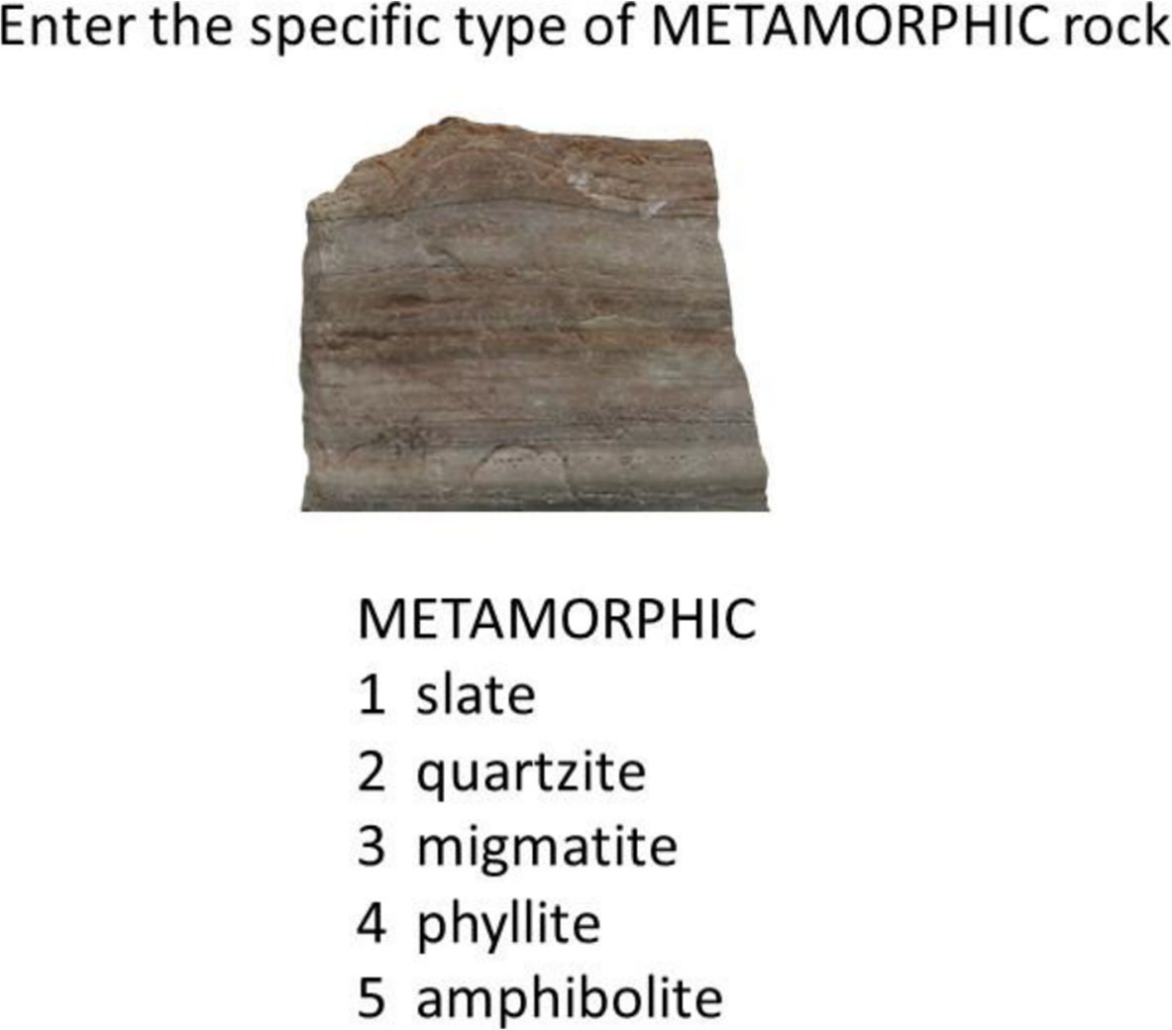
Schematic example of the second stage of a typical trial from the training and test phases of condition 2 and the training phase of condition 3. The example assumes that the subject selected the high-level response “Metamorphic” during the first stage

In condition 2, participants learned simultaneously to classify rocks into both their high-level and subtype-level categories. The condition used a two-stage response procedure. An example screenshot of the first stage of an individual trial is presented in Fig. 3. As illustrated, an individual rock would be presented in the center of the screen. Underneath the rock, the high-level responses igneous, metamorphic and sedimentary were shown in three columns, and beneath each high-level name were shown the subtypes for that high-level category. In the first stage, participants were prompted to enter the high-level response for the rock. Once the high-level response was selected, the second response stage began. As shown in the example screenshot in Fig. 4, the participant was prompted to select the subtype name from among the possibilities for the selected high level. For instance, if a participant had responded that the rock was metamorphic, he or she would then be prompted to select the rock’s subtype name within the metamorphic column. This same two-stage procedure for collecting responses continued to be used in the testing phase of condition 2.

Our central idea in implementing the two-stage response procedure of condition 2 was that it might combine in synergistic fashion various elements of previously tested procedures that have advantageous components. First, because participants are required initially to classify at the high level, they may be motivated to search for features that are diagnostic at that high level. Second, the requirement that participants also learn the subtype-level categories may foster the learning of aspects of the category structure that are disorganized and dispersed (e.g., in which subtypes from contrasting high-level categories are highly similar to one another). Third, the requirement that participants make two separate responses on each individual trial — first the high-level classification response and then the subtype-level one — might be effective in allowing participants to develop learned associations between the high-level and subtype-level names of the rocks.

Nevertheless, in terms of assessing the participants’ acquired knowledge, this experimental condition has the same limitation as did the simultaneous paired-name condition that had been tested in Nosofsky et al.’s (2017) experiment. In particular, because the high-level and subtype-level names were simultaneously present, a participant could in principle focus on only the subtype names during both training and test. On each trial, if the participant decided that a rock was, for instance, ‘granite’, then he or she could enter the corresponding high-level category response (‘igneous’) by making reference to the column in which granite appeared. Thus, an alternative condition was required to evaluate the extent to which the training procedure is effective in allowing participants to directly classify the rocks at the high-level of categorization.

We addressed this requirement by also conducting condition 3. With one exception described below, the training phase for condition 3 was identical to that in condition 2. The key difference across the conditions arose at time of test. Whereas in condition 2 we continued to present the subtypes along with the high-level names at the time of the test (in the column format illustrated in Figs. 3 and 4), in condition 3 the subtypes were no longer presented. Instead, just as in condition 1, the question prompt now made reference to only the high-level categories (as illustrated in Fig. 2). Thus, condition 3 provided a pure test of the participants’ ability to classify the rocks into the high-level categories, without the benefit of an external cue that linked the subtype names to the high-level names.

The second difference between conditions 2 and 3 arose during the training phase. On 80% of the trials, the same two-stage response procedure was used in condition 3 as in condition 2. However, on 20% of the trials, the question prompt was the same as in condition 1; that is, participants were required to classify the rock into one of the high-level categories without the benefit of the external cue showing which subtype names were linked with which high-level names (as in Fig. 2). In addition, on these trials, a reminder message was provided at the bottom of the computer screen stating: “Remember, your primary job is to learn the high-division names.” We included these high-level-only trials to remind participants that their primary task was to learn the high-level name for each rock and to discourage participants from developing a strategy of relying solely on learning the subtype-level names.

**Fig. 5:**
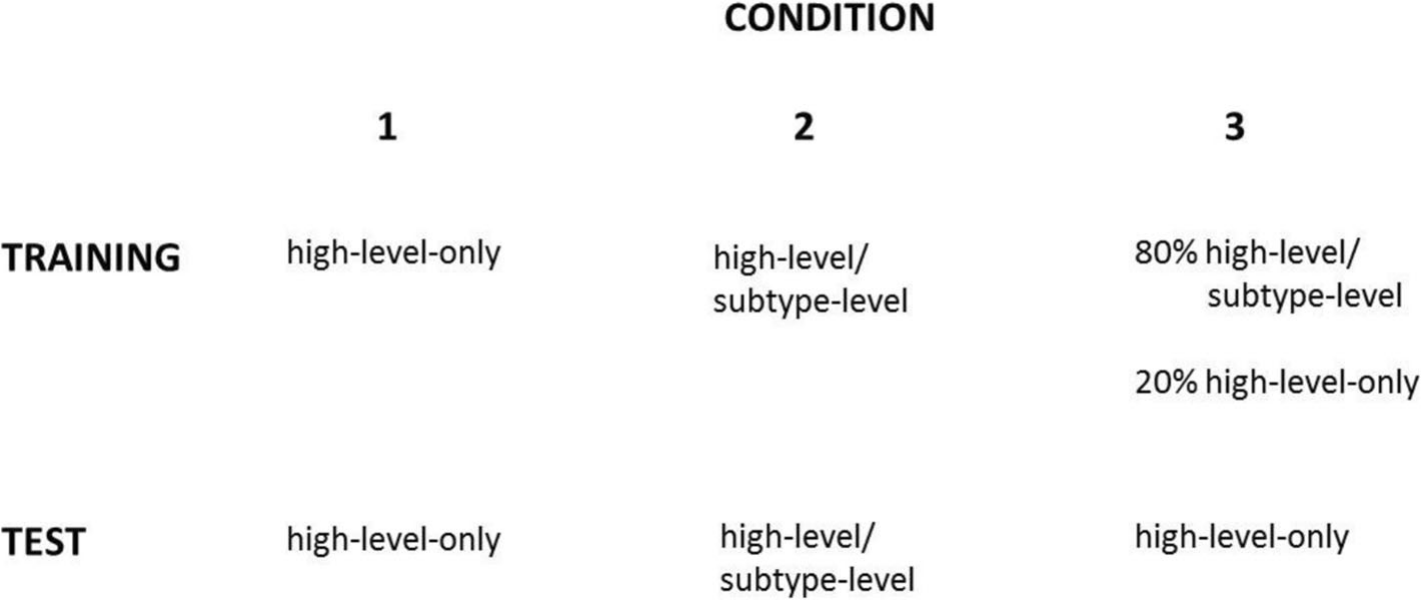
Schematic summary of the training and testing procedures across conditions 1–3. In the high-level-only response mode, participants classified each item into either the igneous, metamorphic or sedimentary high-level categories. In the two-stage high-level/subtype-level mode, participants first made the high-level response, followed by a subtype-level response from among the subtypes that were members of the chosen high-level category

A schematic summary of the training and testing procedures across the three conditions is provided in Fig. 5.

### Method

#### Participants

There were 95 undergraduate students from Indiana University Bloomington who participated as part of a requirement for their introductory psychology courses. The participants all had normal or corrected-to-normal vision and all reported having normal color vision. All reported that they had little or no previous experience in rock classification. Each participant’s condition was randomly assigned, with 32 participants in condition 1, 32 in condition 2, and 31 in condition 3. These sample sizes were as large or larger than in the individual conditions of the closely related studies that most directly motivated the present research and that found significant differences in the outcomes of the broad- versus specific-level training (Miyatsu et al., in press, experiments 1 and 2; Noh et al., 2014; Nosofsky et al., 2017). (As it turned out, the correlation on the repeated old–new item performance measure in our study was *r* = .74; this yielded power = .628 to detect a medium-size main effect of training procedure on test-phase performance, and power = .966 to detect a large-size effect.)

#### Stimuli and apparatus

The stimuli consisted of 360 pictures of rocks from the three broad divisions of igneous, metamorphic and sedimentary rocks. Each broad division comprised 10 subtype categories, listed in Table 1. There were 12 samples of each subtype. The rock picture samples were taken from a variety of online sources (for a fuller description of these stimulus materials, see Nosofsky, Sanders, Meagher, & Douglas, 2018). The experiment was programmed in MATLAB using Psychophysics toolbox (Brainard, 1997) on a personal computer running Microsoft Windows.

#### Procedure

For each individual participant, 5 of the 10 subtypes from each of the three broad divisions of igneous, metamorphic and sedimentary rocks were randomly selected. The participant learned the items from only these randomly selected subtypes. We used this procedure of sampling a subset of the categories because pilot work suggested that overall learning performance would be poor if participants were required to try to learn all 30 categories in a single 1-h session.

In all conditions, the procedure consisted of a training phase and a test phase. The training phase consisted of three training blocks with 90 trials in each block, whereas the test phase consisted of one block with 120 trials. The test phase included presentations of old items from the training phase as well as novel transfer items from the studied categories. Across all conditions, for each individual participant, the members of each rock subtype were randomly assigned as either training or novel transfer stimuli. For each subtype, there were six randomly chosen training examples and four randomly chosen transfer examples.

Across all conditions in the training phase, on each trial, a picture of a training rock was displayed in the center of the screen and the participant attempted to classify it. Each of the individual training items was presented once per block, with the order of presentation of the 90 training items randomized. An analogous procedure was used across all conditions in the test phase. The tested stimuli consisted of four of six randomly selected training examples from each of the 15 subtype categories, and of the four novel transfer items from each of the 15 subtype categories, for a total of 120 test items. The order of presentation of the 120 items was randomized.

The nature of the training and test procedures in each of the conditions has already been described in our introduction to this experiment; here we provide only some additional methodological details. First, in cases in which participants were required to classify the rocks into their high-level categories, they did so by pressing the “i” key for igneous, “m” for metamorphic and “s” for sedimentary. In cases in which participants were required to indicate the subtype category of the rock, they did so by pressing a number on the keyboard that preceded the subtype name on the computer screen (as illustrated in Fig. 4). During the training phase, the computer displayed corrective feedback at the end of each trial (with the rock picture remaining on screen), stating that the participant was either “correct” or “incorrect” followed by the correct response. In condition 1 (and in the 20% of trials in condition 3 that required only a high-level response), the corrective feedback was with respect to only the high-level category of the rock (for example, “Correct! Igneous” or “Incorrect: Sedimentary”). In condition 2 (and in the 80% of trials in condition 3 that used the two-stage response procedure), the corrective feedback was provided after both responses were made. The computer provided simultaneous feedback at both levels, such as “Correct! Igneous-gabbro” or “Incorrect: Metamorphic-marble”. In all conditions, the feedback remained on the screen for 1 s following correct responses and for 2 s following incorrect responses (with the picture of the rock remaining on the screen). The feedback was followed by a 0.5-s inter-trial interval consisting of a blank screen. At the end of each training block, the computer reported to the participants their overall percentage of correct responses. The methodological details of the test phase were the same as already described for the training phase, except no corrective feedback was provided during the test-phase trials. Instead, the computer simply displayed a message of “okay” to indicate to the participants that their response had been recorded. At the end of the testing phase, the participants were thanked and were provided with a debriefing of the purpose of the experiment. The experimental session lasted roughly 50 min.

### Results

#### Training

**Fig. 6:**
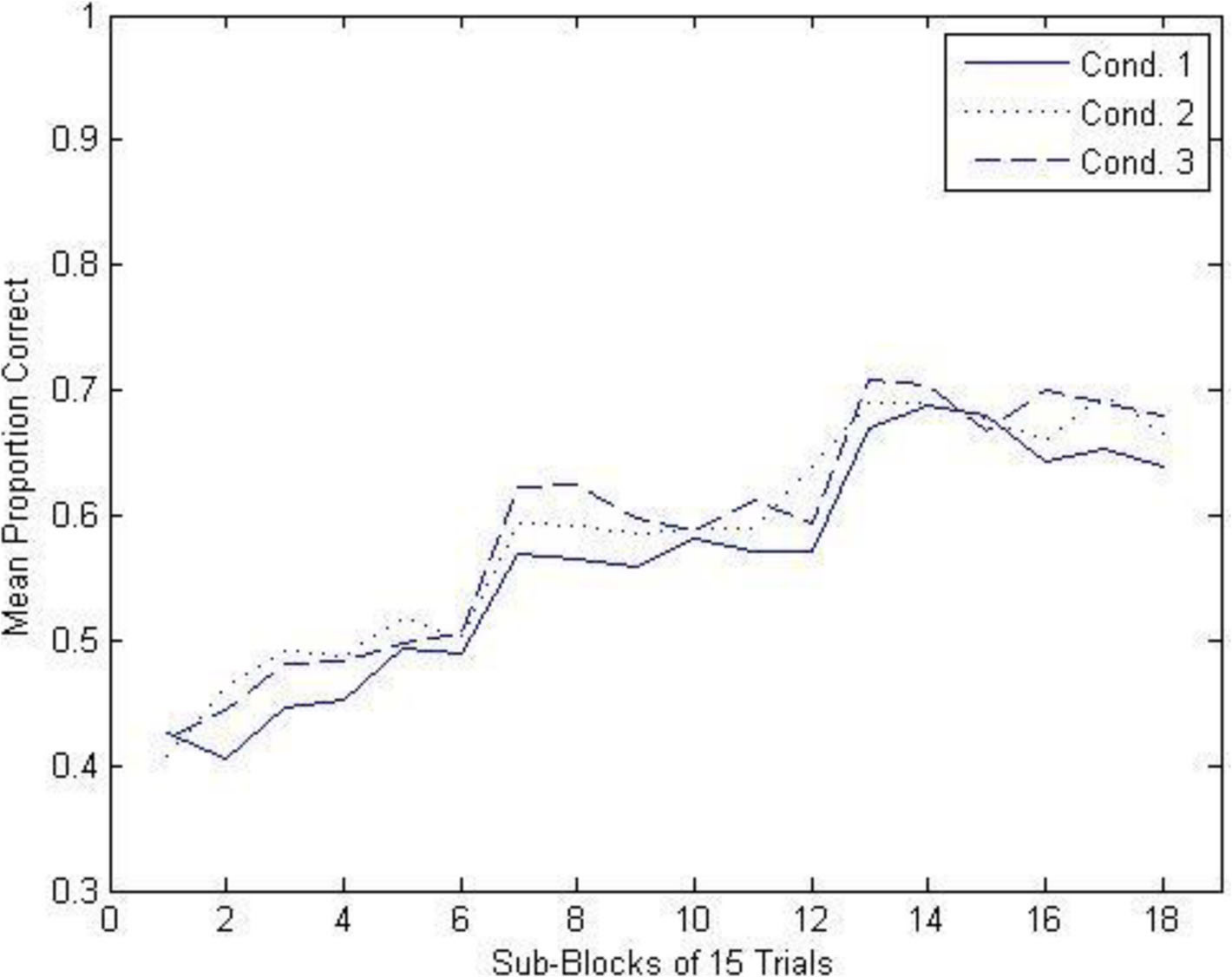
Experiment 1A: mean proportion of correct high-level classification responses as a function of conditions and sub-blocks during the training phase. Cond. condition

To analyze the results from the training phase, we divided the complete sequence of 270 trials into 18 consecutive 15-trial sub-blocks and then measured the mean proportion of correct high-level responses in each sub-block. The results for each of the three conditions are shown in Fig. 6. These data were submitted to a 3 × 18 mixed model analysis of variance (ANOVA), with condition (1, 2, 3) as the between-subjects variable and sub-block (1–18) as the within-subjects variable. As can be seen, overall performance improved dramatically in all three conditions as a function of training, *F* (17, 1564) = 49.014, *MSE* = .016, *p* < .0001; the degree of improvement in performance did not significantly vary across conditions, *F* (34, 1564) = .445, *p* = .998 for the interaction. Thus, not surprisingly, our naive participants did not enter the experiment with significant amounts of prior knowledge of the high-level rock category assignments, but rather learned these assignments during the course of the training phase.

Importantly for present purposes, there was no significant difference in high-level naming performance as a function of condition, *F*(2, 92) = .934, *MSE* = .148, *p* = .397. Thus, requiring participants to make a subtype-level classification following the high-level classification during training (conditions 2 and 3) did not negatively impact learning of the high-level classification of the training items relative to focusing participants only on high-level classification (condition 1); indeed, if anything, learning of high-level classifications was slightly better when subtype classification also had to be learned (see Fig. 6).

#### Test

**Fig. 7:**
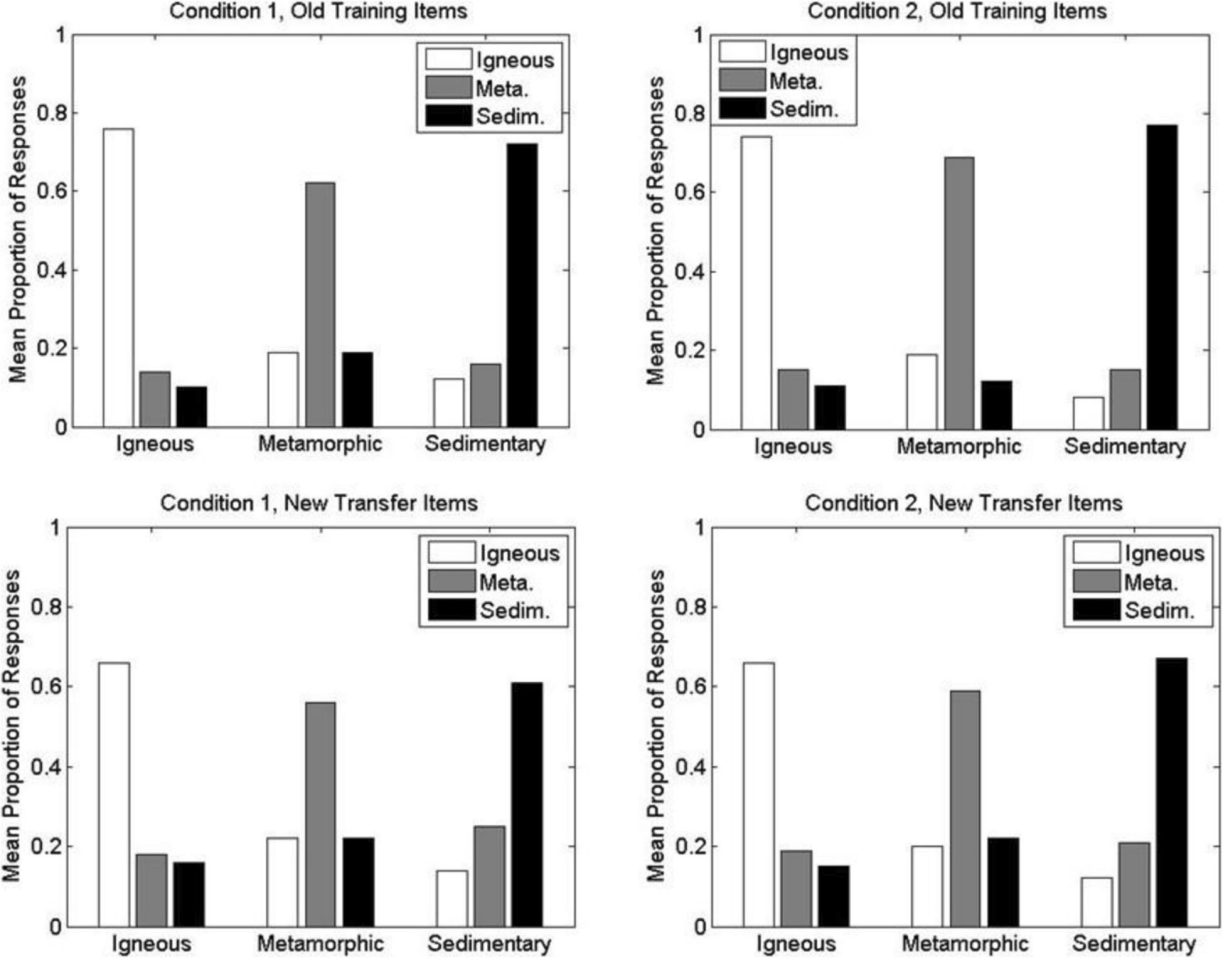
Experiment 1A: mean proportion with which members of each high-level category were classified into the alternative high-level categories during the classification test phase of conditions 1 and 2. (Left panels) Condition 1; (right panels) condition 2; (upper panels) old training items; (lower panels) new transfer items. For example, within each panel, the white bar to the far left indicates the mean proportion with which members of the high-level igneous category were correctly classified as igneous, whereas the adjacent gray bar indicates the mean proportion with which members of the igneous category were incorrectly classified as metamorphic (Meta.). Sedim. sedimentary

**Fig. 8:**
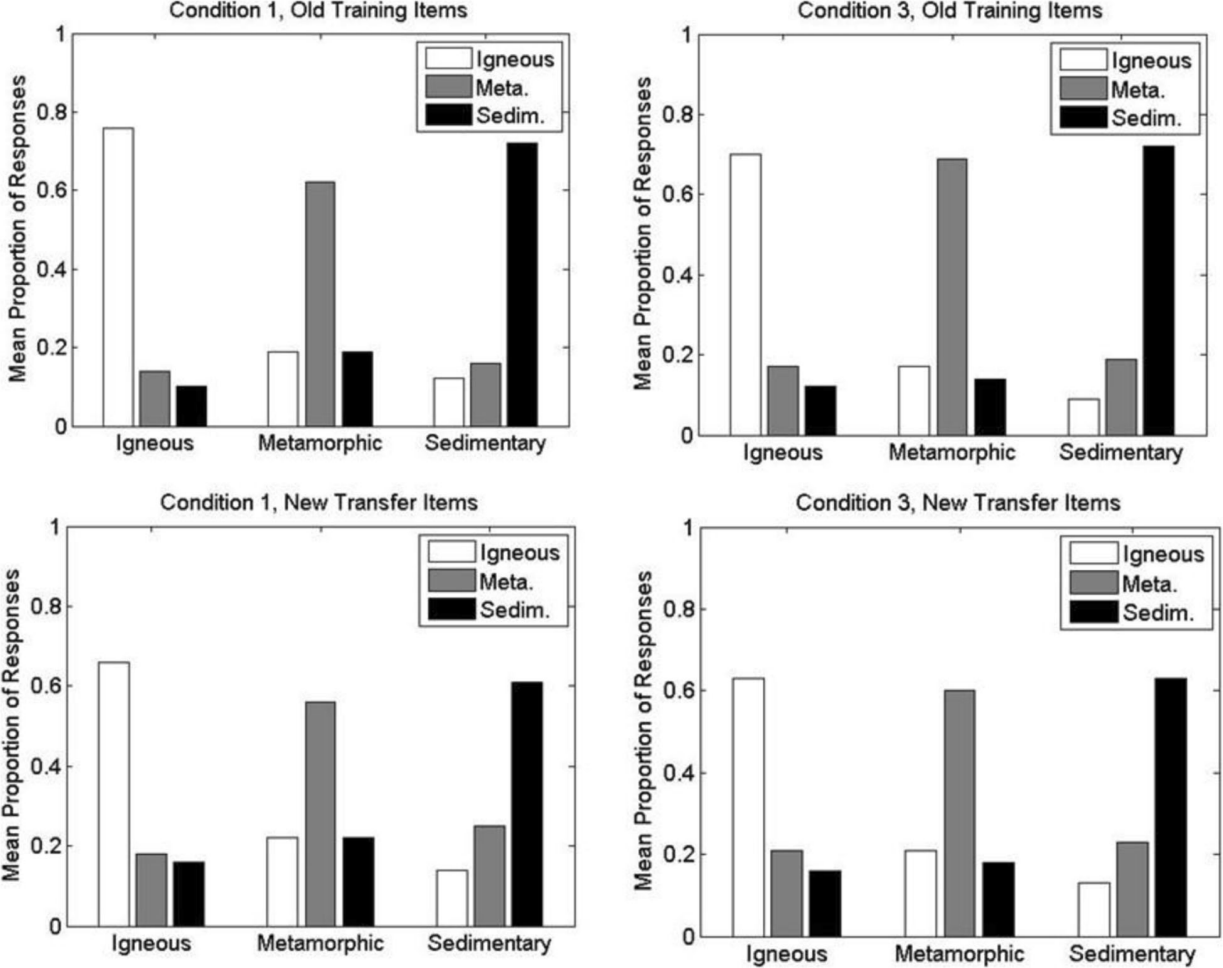
Experiment 1A: mean proportion with which members of each high-level category were classified into the alternative high-level categories during the classification test phase of conditions 1 and 3. (Left panels) Condition 1; (right panels) condition 3; (upper panels) old training items; (lower panels) new transfer items. For example, within each panel, the white bar to the far left indicates the mean proportion with which members of the high-level igneous category were correctly classified as igneous, whereas the adjacent gray bar indicates the mean proportion with which members of the igneous category were incorrectly classified as metamorphic (Meta.). Sedim. sedimentary

The results from the test phase are shown in Figs. 7 and 8. The figures show the mean proportion with which participants classified members of each main high-level division of rocks (igneous, metamorphic, sedimentary) into each of the high-level divisions. Within each figure, the top panels show the results for the old training items whereas the bottom panels show the results for the new transfer items. For ease of comparison, Fig. 7 shows performance for condition 1 (left panels) versus condition 2 (right panels), whereas Fig. 8 shows performance for condition 1 (left panels) versus condition 3 (right panels).

Inspection of the results reveals that participants classified the rocks into their correct high-level categories well above chance levels (.33) in each of the conditions. In addition, as would be expected, correct classification performance for the old training items presented during the test phase was higher than for the novel transfer items. The pattern of results was also fairly similar across each of the three main divisions of rocks (igneous, metamorphic and sedimentary).

The key question concerns the comparisons of performance across the different training and testing conditions. Participants in condition 2 achieved nominally higher correct proportions than did participants in condition 1 on both the old training items (condition 1, *M*_*1*_ = .70, *SD*_*1*_ = .14; condition 2, *M*_*2*_ = .73, *SD*_*2*_ = .12) and the new transfer items (condition 1, *M*_*1*_ = .61, *SD*_*1*_ = .12; condition 2, *M*_*2*_ = .64, *SD*_*2*_ = .10). Participants in condition 3 performed virtually the same as did participants in condition 1 on both the old training items (*M*_*1*_ = .70; *M*_*3*_ = .70, *SD*_*3*_ = .15) and the new transfer items (*M*_*1*_ = .61; *M*_*3*_ = .62, *SD*_*3*_ = .13). We analyzed these data with a 3 × 2 mixed-model ANOVA, with condition (1–3) as the between-subjects factor and item type (old versus new) as the within-subjects factor. Old items were classified significantly more accurately than new items, *F*(1, 90) = 93.91, *MSE* = .004, *p* < .001. However, there was no significant effect of condition, *F*(2, 90) = 0.46, *MSE* = .030, *p* = .63, and no significant interaction between condition and item type, *F*(2, 90) = 0.17, *MSE* = .004, *p* = .85.

### Discussion

In their previous study, Miyatsu et al. (in press) found that direct high-level-only training led to significantly better high-level classification performance at the time of the test than did training in which participants were required to also learn the subtype-level names of the rocks (see also Noh et al., 2014). If anything, our results go slightly in the opposite direction, with performance in the simultaneous paired-name conditions being at least as good as performance in the high-level-only condition. Importantly, this pattern held even in the paired-name training condition in which participants were no longer provided with the subtype names of the rocks at the time of transfer. However, before discussing the most likely reasons for these contrasting results, we first report a second study to confirm the reliability of our findings. Our tentative conclusion from the present experiment is that the present form of paired high-level/subtype-level training leads to no disadvantage in high-level naming performance compared to the case in which participants are trained at only the high level. Because the conclusion rests on a finding of no difference (i.e., a null result), a possible concern is that forms of experimental noise could be hiding a high-level-only advantage. One such potential form of experimental noise is that each participant in experiment 1A was exposed to randomly selected subsets of rock subtypes from each of the high-level categories. If, by happenstance, participants in the paired-name condition were exposed to an easier set of subtypes, then the results could be hiding what is a true high-level-only advantage. We conducted experiment 1B to address this possibility.

## Experiment 1B

Experiment 1B was the same as experiment 1A, with the following exceptions. First, because the central comparison of interest involves the conditions that evaluate high-level naming performance in cases in which subtype names are not provided at the time of the test, we omitted condition 2 from the design. Second, the stimulus materials were assigned to individual participants across conditions 1 and 3 in a yoked fashion. The subtypes, training items and transfer items were chosen randomly for each individual participant *i* in condition 1, using the same constraints as described in experiment 1A. Those same subtypes, training and transfer items were then assigned to a matched participant *i* in condition 3. Finally, to gain some additional information concerning the results of the training procedure, we tested participants in a paired-associate naming task. On each trial of the task, one of the subtype-level names was presented, and participants chose which of the three high-level names with which it was associated.

### Method

#### Participants

The participants were 102 undergraduate students from Indiana University Bloomington who received credit towards a requirement for their introductory psychology courses. Again, all participants had normal or corrected-to-normal vision, reported having normal color vision, and reported little or no previous experience in rock classification. In sequential fashion upon arrival to the laboratory, half the participants were assigned to condition 1 and half to condition 3. Thus, there were 51 participants in condition 1 and 51 in condition 3. We used this increased sample size with the goal of increasing the statistical power from experiment 1a. (As it turned out, the correlation on the repeated old–new item performance measure in experiment 1b was *r* = .86; this yielded power = .737 to detect a medium-size main effect of training procedure on test-phase performance, and power = .986 to detect a large-size effect.)

#### Stimuli and apparatus

The stimuli and apparatus were the same as in experiment 1A.

#### Procedure

The method of choosing subtypes, training stimuli and transfer stimuli for the condition 1 participants was the same as in experiment 1A. For each participant *i* in condition 1, there was a yoked participant *i* in condition 3. Each yoked participant received the same subtypes and training and transfer stimuli as did the corresponding condition 1 participant.

All other aspects of the experiment 1B procedure were the same as in experiment 1A, except for the inclusion of a paired-associate naming test in experiment 1B. The paired-associate test was conducted following the classification training phase but prior to the classification transfer phase. The names of the 15 subtypes experienced by each participant were presented in random order, one per trial. For each subtype name, the participant indicated whether it was a member of the igneous, metamorphic or sedimentary categories by pressing the “i”, “m” or “s” response keys, respectively. No feedback was provided during this test. The participants from both conditions engaged in the paired-associate task, despite the fact that those in condition 1 had received no training on classifying the rocks into the subtype categories. Thus, the condition 1 participants served as a source of comparison for evaluating the learning of the name associations of the condition 3 participants.

### Results

#### Training

**Fig. 9:**
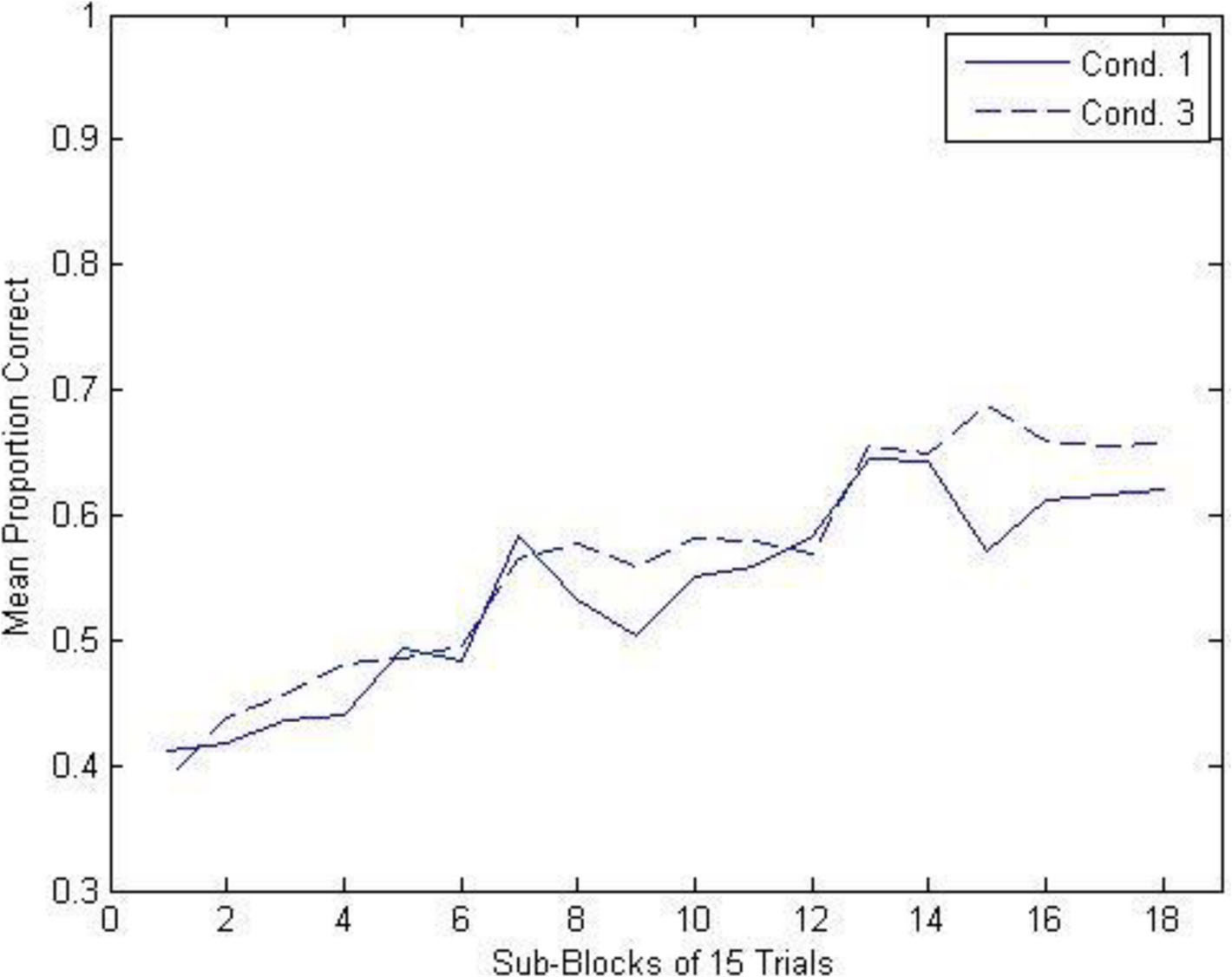
Yoked experiment 1B: mean proportion of correct high-level classification responses as a function of conditions and sub-blocks during the training phase. Cond. condition

The training results are shown in Fig. 9. The training results show the same pattern as in experiment 1A. High-level naming performance increased dramatically across the training session, *F* (17, 1700) = 39.739, *MSE* = .017, *p* < .0001, and the pattern of the increase did not significantly differ across the two conditions, *F* (17, 1700) = 1.571, *p* = .064 for the interaction. The key finding was that requiring classification of both the high-level category and the subtype-level category (on 80% of the trials in condition 3) did not significantly alter the performance level of high-level classification learning relative to requiring only high-level classification during training (condition 1), *F* (1, 100) = 1.573, *MSE* = .173, *p* = .213.

#### Naming associative learning

In the paired-associate naming test, the high-level naming (condition 1) participants scored 40.4% correct on average, which is only slightly better than chance (33.3%) as would be expected. By contrast, the condition 3 participants scored 85.5% correct, a substantial and significant improvement over that in condition 1, *F* (1, 100) = 330.38, *MSE* = .016, *p* < .0001 (analysis on proportion correct). Thus, although the condition 3 participants never received direct paired-associate name training, the two-stage response procedure appears to have been reasonably effective in allowing most of them to learn the name associations. Indeed, approximately half of these participants (25) learned from 93 to 100% of the associations between high-level category names and their corresponding subtype category names.

#### Test

**Fig. 10:**
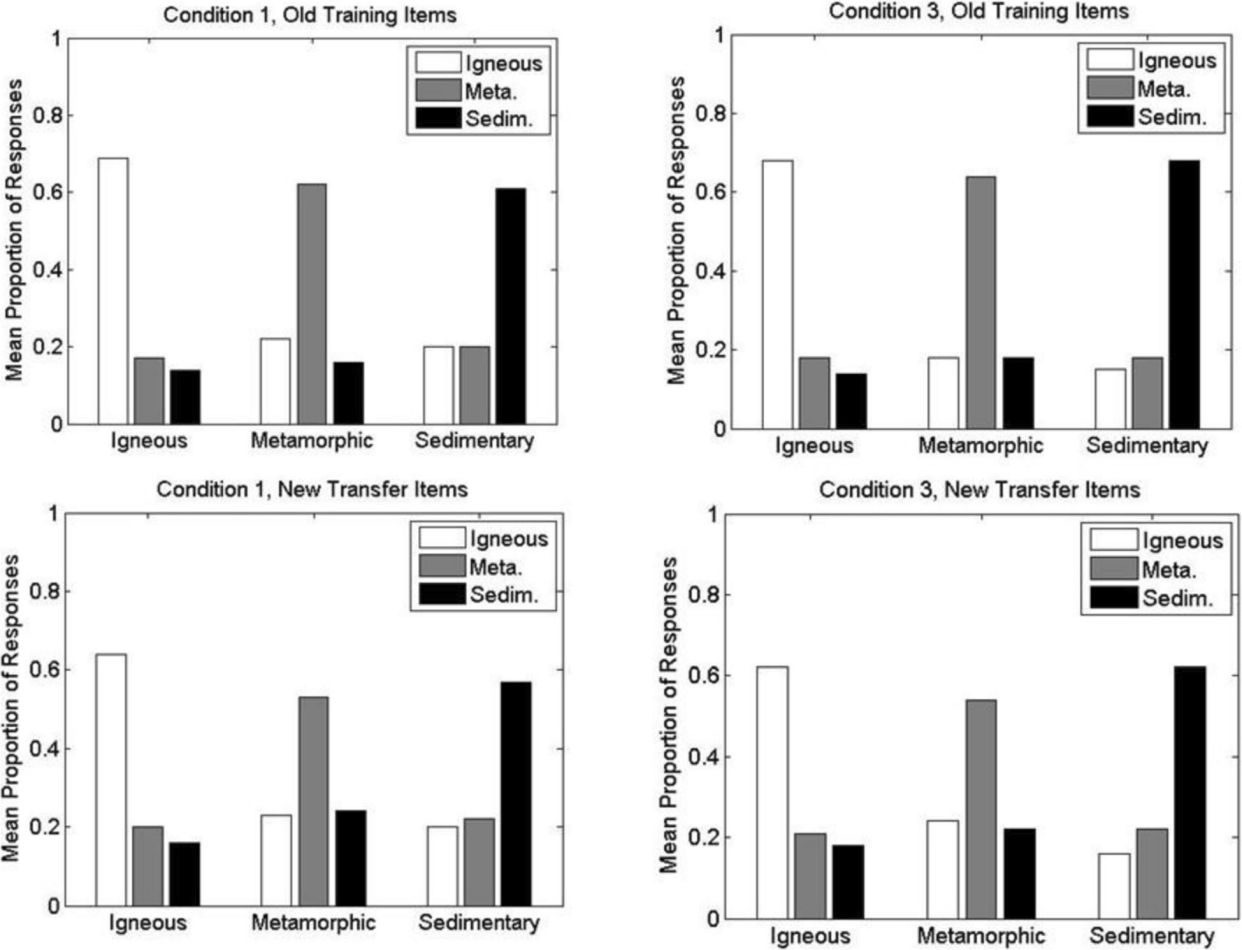
Yoked experiment 1B: mean proportion with which members of each high-level category were classified into the alternative high-level categories during the classification test phase of conditions 1 and 3. (Left panels) Condition 1; (right panels) condition 3; (upper panels) old training items; (lower panels) new transfer items. For example, within each panel, the white bar to the far left indicates the mean proportion with which members of the high-level igneous category were correctly classified as igneous, whereas the adjacent gray bar indicates the mean proportion with which members of the igneous category were incorrectly classified as metamorphic (Meta.). Sedim. sedimentary

The results from the rock classification test phase are shown in Fig. 10. The pattern of results is similar to those seen in experiment 1A. Averaged across the high-level categories, participants in condition 3 achieved slightly higher correct proportions than did participants in condition 1 on both the old training items (condition 1, *M*_*1*_ = .64, *SD*_*1*_ = .15; condition 3, *M*_*3*_ = .67, *SD*_*3*_ = .16) and the new transfer items (condition 1, *M*_*1*_ = .58, *SD*_*1*_ = .14; condition 3, *M*_*3*_ = .59, *SD*_*3*_ = .14). A two-factor mixed-model ANOVA indicated that the slight differences across conditions 1 and 3 were not significant, *F*(1, 100) = 0.42, *MSE* = .040, *p* = .52, and did not interact with item type, *F*(1, 100) = 0.80, *MSE* = .003, *p* = .37. As in experiment 1a, there was significantly better performance on old than new items, *F*(1, 100) = 75.43, *MSE* = .003, *p* < .001.^3^

## General discussion

The purpose of this study was to pursue issues related to the teaching of hierarchically organized science categories. The example target domain was the teaching of rock categories in the geologic sciences. The main question was how the teaching of high-level category names for rocks (igneous, metamorphic, sedimentary) would be influenced by requiring participants to simultaneously learn the rocks’ subtype-level names (e.g., granite, marble, shale). Past research that examined this question in both the rocks domain and related domains found varying patterns of results (e.g., Lassaline et al., 1992; Miyatsu et al., in press; Noh et al., 2014; Palmeri, 1999; Verheyen et al., 2008; for research examining a related question, see Tanaka, Curran, & Sheinberg, 2005).

Nosofsky et al. (2017) suggested and found support for the hypothesis that the effectiveness of the high-level teaching strategy may vary with the precise structure of the to-be-learned categories. High-level-only training may be more effective in cases in which the high-level categories occupy compact regions in a multidimensional similarity space; however, simultaneous high-level/subtype-level training may be more effective in cases in which the high-level category structures are disorganized and dispersed. The practical implications of Nosofsky et al.’s findings, however, are unclear. First, these researchers constructed the compact and dispersed structures by selectively sampling rock subtypes from the high-level categories; the relevance to the teaching of more authentic categories, such as those taught in college-level introductory geology courses, is unknown. Second, in the condition that used simultaneous high-level/subtype-level training, the subtype name was always available to the participants to guide their responses. It is unknown whether participants could have produced the correct high-level names in the absence of the simultaneously present subtype names.

Miyatsu et al. (in press) pursued these issues in two main ways. First, rather than selectively sampling rock subtypes to produce either compact or dispersed structures, these researchers conducted a more random sampling by using the availability of pictures of particular rock types from web searches as a proxy for how often the rocks appear in educational and recreational situations. In addition, in their experiments of most practical relevance to the present question, participants were tested on their ability to directly classify into the high level (without the benefit of the subtype names being present). In brief, Miyatsu et al. found that participants who were required to learn to classify at the subtype level performed significantly *worse* at high-level classification than did groups that focused solely on learning the high-level names (experiments 1 and 2). As we reviewed in our introduction, however, there were various limitations associated with Miyatsu et al.’s training procedures. One limitation is that at least some participants likely formed relatively weak associations between the subtype-level and high-level names. Hence, even if they learned to classify extremely accurately at the subtype level, they would be severely impaired when asked to provide category names at the high level. A second limitation is that, in the condition in which participants first learned to classify at the subtype level and then received paired-associate training between the subtype-level and high-level names, the participants never received any direct training at the high level. Thus, they never had the opportunity to learn any direct associations between the rocks and the high-level categories, nor to learn to attend to any features that were diagnostic for classifying items at the high level.

The key idea that we pursued in the present research was to implement a two-stage response-training procedure that involved simultaneous training at both the high and subtype levels and that would potentially address the limitations noted above. On each trial, for each individually presented rock sample, a participant would first indicate a high-level category response, followed by a subtype-level response. Feedback was then presented simultaneously at both levels of the hierarchy. Our hypothesis was that this procedure might combine effective elements of the alternative training techniques in a synergistic fashion. First, asking for high-level responses might motivate participants to learn to attend to features of the rocks that are diagnostic of the high-level categories and to form some direct associations between the rocks and their high-level classifications. Second, asking for subtype-level responses might be effective in guiding the learning of aspects of the category structure that are disorganized and dispersed. Third, the two-stage procedure might be effective in allowing participants to develop learned associations between the high-level and subtype-level names of the rocks. Thus, participants would be well positioned to transfer their knowledge to a testing situation that required direct classification into the high-level categories.

Briefly, we found that participants trained using this two-stage high-level/subtype-level procedure showed high-level classification performance that was at least as good as a group that was trained solely at the high level. Furthermore, we observed the result in an experimental situation in which participants learned to classify a broad range of subtypes of rocks that are highly representative of those taught in introductory college-level geology courses. In addition, the result was observed in conditions in which the subtype name was no longer present to help guide the participants’ high-level classification response.

Admittedly, in the present experiments, requiring participants to simultaneously learn at the subtype level did not significantly *improve* their high-level classification performance (compared to a high-level-only group). The more important take-home message, however, is that, contrary to the results reported in Miyatsu et al. (in press, experiments 1 and 2), it certainly did not hinder their high-level learning. This result suggests that, in a setting in which students are tasked with learning high-level names for authentic sets of igneous, metamorphic and sedimentary rocks that are commonly taught in introductory college-level geoscience classes, it is best for students to learn simultaneously at both the high and subtype levels (using training techniques similar to the presently investigated one). The obvious reason is that students are learning far more total information. First, in addition to learning the high-level names for the rocks, they are also learning their subtype names. In this regard, it is important to note that the kinds of reasoning and inferencing for which geologists rely on rock type categorization often depend on making accurate subtype classifications of rock samples. As noted in the introduction, classification of an igneous rock as either rhyolite or granite allows fine-grained inferences about the history of a terrain. As such, in the introductory geosciences courses of which we are aware, the learning objectives include learning both the high-level and the subtype-level classifications of rocks. From an instructional standpoint, the present simultaneous training procedure provides an efficacious technique for doing so.

Second, the paired-associate name-test results (experiment 1b) clearly showed that the two-stage high-level/subtype-level training also supported good learning of the hierarchy of classification levels for rocks; that is, participants in this training group learned well the subtype classifications that belong to each high-level category. In comparison, Miyatsu et al. (in press, experiment 2) implemented a name-learning study phase in which participants were presented with the high-level–subtype-level name pairings, followed by three test-feedback trials. This name-learning phase decreased the total amount of rock classification training the participants received, but still produced only 76% accuracy at knowing the name pairings. Accordingly, the current two-stage training procedure is attractive because it supports more accurate name-pair learning (86%) with no reduction in rock classification training.

A potential concern regarding our suggestion is that participants using the two-stage response procedure are spending slightly greater time on the task than are participants who use the high-level-only response procedure. On each trial, the participants using the two-stage procedure make two key presses, whereas those using the high-level-only procedure make only a single key press. (Importantly, however, total response–feedback time was held constant across the two conditions.) In our view, this slightly greater time-on-task is a minor consideration given the dramatic gains in overall category knowledge that are achieved.

There are a number of limitations of the present study that need to be pursued in future work. First, we investigated participants’ ability to generalize to new members of the categories only in a test that occurred immediately following initial training. An important question is whether the same effects would also be observed in delayed tests. Second, although we argued in our introduction that learning of rock categories is crucial to inference and causal reasoning in the geologic sciences, it is important to demonstrate that the effects of our proposed two-stage training procedure do indeed extend beyond mere classification accuracy to various forms of geologic problem-solving as well. Third, the present study did not involve classification of real, physical rocks; instead, it was limited to the use of only rock images. In general, we would expect that our two-stage training procedure would yield similar patterns of results regardless of whether the to-be-classified materials are images or physical rocks. Nevertheless, this hypothesis also needs to be pursued in future research.^4^

Much work remains for future investigations of the training techniques that might be most effective in teaching students to classify objects into hierarchically organized natural science categories. For example, Meagher, Carvalho, Goldstone, and Nosofsky (2017) found evidence that the use of organized, simultaneous visual displays provides a promising technique for conveying the hierarchical structure of rock categories. Much other research has also pursued techniques for effectively teaching categories at a single level of a hierarchy. Such techniques include ones that manipulate the presentation sequences of training instances from contrasting categories (e.g., Carvalho & Goldstone, 2014; Eglington & Kang, 2017; Kornell & Bjork, 2008; Mathy & Feldman, 2016); the order in which hard versus easy instances are presented (e.g., Pashler & Mozer, 2013); the size of the sets of training instances and which specific training instances to use (e.g., Nosofsky et al., 2018, 2019; Wahlheim et al., 2012); and the use of explicit coaching such as visual highlighting of diagnostic features (Miyatsu, Gouravajhala, Nosofsky, & McDaniel, 2019). Combining these techniques with variants of the presently proposed two-stage response procedure might yield even better learning of hierarchically organized science categories.

## Data Availability

All data and materials are available from the first author upon request. If the article is published in the journal, all data and materials will be made publicly available on the Open Science Framework.

## Footnotes

1 Miyatsu et al. (in press) reported two additional experiments (experiments 3 and 4) in which participants were trained and tested at only the subtype level; however, analogous to the scoring method used by Nosofsky et al. (2017), a response was scored as correct if the indicated subtype was a member of the correct high-level category. In these experiments, Miyatsu et al. observed no significant difference in overall performance across the high-level-only and subtype-level training conditions. One possible interpretation is that the category structure tested by Miyatsu et al. was intermediate on the compact–dispersed continuum, so neither category-teaching procedure had a significant advantage. Alternatively, the simultaneous paired-naming procedure that had been used by Nosofsky et al. may have fostered learning at *both* levels of the hierarchy; because Miyatsu et al. provided no training at the high level in the subtype-training conditions of their experiments 3 and 4, participants may not have learned to attend to features of the rocks that were diagnostic for high-level naming. Even if participants had achieved improved high-level scores in the subtype-naming conditions of Miyatsu et al.’s experiments 3 and 4, the practical implications would still be limited: participants would have achieved zero knowledge of the actual high-level names associated with the subtype names.

2 Whereas Miyatsu et al. (in press) also tested a broad battery of rock subtypes from the igneous, metamorphic and sedimentary categories, a significant subset of them are not commonly taught in college-level introductory geoscience classes. Hence, in terms of the to-be-learned materials, the present study has more direct relevance to authentic, introductory college-level teaching situations.

3 Because participants across the two conditions were yoked by stimulus items, we also conducted a 2 × 2 repeated measures ANOVA with condition (and item type) as a within-subject factor. The results were identical to the mixed ANOVA with condition as a between-subjects factor (*F* values but not error terms deviated very slightly). This is as expected because the yoking by stimulus items produced negligible correlation in performance between conditions (e.g., for old item, *r* = .053). The same held when the paired-associate name results were analyzed with a repeated-measure ANOVA (with condition as the repeated measure).

4 Meagher, Cataldo, Douglas, McDaniel, and Nosofsky (2018) argued and found support for the hypothesis that optimal procedures for training real-world rock classifications would likely combine the use of physical samples and image-based teaching technology. One reason is that the use of computer images provides an efficient means to allow students to experience the extensive within-category variability associated with most rock categories, something not easily achieved with techniques that rely solely on the use of actual physical samples. Indeed, a potential limitation of what appears to be ‘standard practice’ in many introductory, college-level geoscience courses is reliance on the use of single physical samples for the purposes of training for each subtype category. Our informal discussions with geoscience instructors suggest heavy reliance on a ‘rocks in a box’ approach. In this approach, students are provided with a box containing a single, prototypical rock sample from each of the main subtypes of the high-level division that is being studied at that point in the class schedule. The students then use instructor-described or laboratory manual procedures to practice identifying each individual rock in the box. In these approaches, students are rarely exposed to the enormous variability in individual rock samples that compose each of the to-be-learned categories in the real world. But there is clear evidence from the cognitive psychology of teaching categories that exposure to variable training instances fosters observers’ ability to generalize to novel transfer items (e.g., Homa & Vosburgh, 1976; Nosofsky, Sanders, Zhu, & McDaniel, 2019; Posner & Keele, 1968; Wahlheim, Finn, & Jacoby, 2012).
